# Cost-effectiveness of the dual prevention pill for contraception and HIV pre-exposure prophylaxis

**DOI:** 10.3389/frph.2023.1144217

**Published:** 2023-05-17

**Authors:** Masabho P. Milali, Danielle Resar, David Kaftan, Jennifer Campbell, Adebanjo Olowu, Danny Edwards, Ingrida Platais, Hae-Young Kim, Sarah Jenkins, Anna Bershteyn

**Affiliations:** ^1^ Department of Population Health, NYU Grossman School of Medicine, New York, NY, United States; ^2^ Clinton Health Access Initiative, Boston, MA, United States

**Keywords:** dual prevention pill, prEP, oral contraception pill, HIV, cost-Effectiveness, daly, hiv prevention, multipurpose prevention technologies

## Abstract

**Introduction:**

Women in sub-Saharan Africa (SSA) experience the world's highest rates of both HIV infection and unintended pregnancy. The Dual Prevention Pill (DPP) is a novel multipurpose prevention technology (MPT) that co-formulates HIV pre-exposure prophylaxis (PrEP) and combined hormonal oral contraception into a single daily pill. As a dual indication product, the DPP may be preferred by women facing these overlapping health risks. However, most SSA countries face severe healthcare resource constraints. Research is needed to assess whether, in what populations, and in what use cases the DPP would be cost-effective.

**Methods:**

We augmented an agent-based SSA HIV model with maternal health parameters including unintended pregnancy, abortion, and maternal mortality. Based on a previous market analysis, we assumed a primary DPP user population of current oral contraceptive users ages 25–49, and alternative user populations in different risk groups (age 15–24, sex workers, HIV-serodiscordant couples) and baseline product use profiles (unmet need for contraception, oral PrEP use, condom use). In three geographies (western Kenya, Zimbabwe, South Africa), we estimated HIV infections averted, pregnancies averted, disability-adjusted life-years (DALYs) averted, and the incremental cost-effectiveness ratio (ICER) over a 30-year time horizon, assuming equivalent adherence to the DPP as to oral contraceptives, higher adherence, or lower adherence.

**Results:**

The DPP is likely to be a cost-effective alternative to oral PrEP among users in need of contraception. Among women not already using PrEP, the DPP is likely to be cost-saving in sex workers and serodiscordant couples. The DPP is unlikely to be cost-effective in oral contraceptive users in the general population. Switching from oral contraception to the DPP could be net harmful in some settings and populations if it were to substantially reduces adherence to oral contraception. Results were robust to a range of time horizons or discount rates.

**Conclusion:**

The DPP has the potential to be cost-effective and cost-saving in populations at substantial HIV risk. Outcomes are sensitive to adherence, implying that effective counseling and decision-making tools for users considering the DPP will be essential. More research is needed to understand real-life adherence patterns and ensure health benefits achieved from contraception alone are not lost.

## Introduction

In 2019, HIV/AIDS was the leading cause of death while pregnancy and delivery complications were the second-leading cause of death among women of reproductive age in sub-Saharan Africa (SSA) (1). Women in SSA experience the world's highest rates of HIV infection (2–4) and of unintended pregnancy (3, 4). In 2021, women and girls accounted for 63% of all new HIV infections in SSA, with over 540,000 new HIV infections in total (5). Meanwhile, the unintended pregnancy rate in SSA is 91 per 1,000 women aged 15 to 49, the highest of any region (6).

While oral pre-exposure prophylaxis was first approved by the US Food and Drug administration (FDA) in 2012, availability and uptake of oral pre-exposure prophylaxis (PrEP) for HIV prevention among women in SSA has been low due to limited funding for the HIV response and slow, relatively fragmented rollout experiences in many countries. Further, the impact of oral PrEP has been hindered by low adherence and continuation rates due to a range of challenges at the structural, community, and individual level, including PrEP stigma and pill burden (7–9). Meanwhile, in SSA efforts to satisfy unmet need for contraception have also struggled to expand, with <1% growth in modern contraceptive prevalence (MCPR) since 2017, compared to more rapid growth in the decade prior (10).

These statistics suggest a need for additional prevention options to meet the diverse needs and preferences of women facing dual health risks of HIV and unintended pregnancy. Multipurpose prevention technologies (MPTs) are products that provide protection from two or more reproductive health issues, including unintended pregnancy, HIV, and other sexually transmitted infections. Currently, the only available MPTs for HIV and pregnancy prevention are male and female condoms, which are non-discreet, often reliant on partner negotiation, and sub-optimally effective with typical use (11). The Dual Prevention Pill (DPP), which co-formulates the active ingredients of combined hormonal oral contraceptives and oral PrEP into a single daily pill, is likely to be the next MPT to reach markets (12). Because the DPP combines two products that are already widely approved, including by the US FDA, regulatory submissions will leverage evidence from bioequivalence studies, a relatively short development pathway, with possible licensure as early as 2024 (12, 13). Evidence from family planning suggests that use of modern contraception increases when more methods become available, as a wider set of options improves the ability to meet user needs over time (14). As a new method option, the DPP therefore offers the opportunity to expand choice and potentially increase PrEP and/or contraceptive coverage. Multiple preference studies have also found that women, partners, and matriarchs would prefer MPTs over single indication HIV prevention products (15–17).

Despite these potential benefits, future availability of the DPP in SSA is uncertain because most SSA countries face severe healthcare resource constraints and need to make difficult tradeoffs in terms of health care service prioritization. For example, during early introduction of oral PrEP, many countries did not prioritize provision of oral PrEP to women in the general population (i.e., outside of specific high-risk groups such as sex workers) because it was not shown to be cost-effective in this population (18). However, more recent evidence suggests that oral PrEP may be cost-effective for women in the general population in high-incidence areas of SSA, especially if PrEP is concentrated in seasons of risk, such as 3-month periods when women have condomless sex (19). As the DPP development proceeds, SSA health authorities will need evidence on cost-effectiveness to inform DPP introduction and scale-up decision-making, including identification of priority populations and geographies, target-setting, and optimization of HIV prevention and contraception method mixes.

To understand the potential cost-effectiveness of the DPP, we used agent-based modeling of HIV transmission and unintended pregnancy in three SSA countries: Kenya, Zimbabwe, and South Africa. We considered DPP use among current OCP users, who are likely to have the highest demand for the DPP, as well as women with unmet need for contraception or who use condoms for contraception, in whom the DPP could provide a more effective form of contraception. We also considered different risk groups, including female sex workers and women with HIV-positive partners, in whom PrEP was previously shown to be more cost-effective than in other population groups (18). Finally, we considered that DPP adherence may differ from OCP adherence, including potentially lower adherence to DPP compared to OCP due to its larger pill size and potential for additional side effects. This analysis was initially performed to inform DPP development, but could help inform future planning for the availability of the DPP in SSA and may have implications for the development of future MPTs.

## Methods

### Model description

Analyses were conducted using the Epidemiological MODeling (EMOD) software, an agent-based network model of sexual and vertical HIV transmission (20, 21). Sexual HIV transmission is modeled using a network of marital, informal, transitory, and commercial sexual relationships, each with distinct age/sex patterns of formation and dissolution, and vertical transmission is modeled upon live birth by an HIV-positive mother (22, 23). Patterns of HIV prevalence and incidence by age, sex, and over time have been compared to population-based survey data in multiple SSA settings, including successful prospective validation of an HIV incidence prediction in a blinded, multi-country community-randomized controlled trial (21).

### Model fit to settings

We configured the EMOD model to fit demographic and HIV trends in western Kenya, South Africa and Zimbabwe using setting-specific census, fertility, and mortality estimates as well as HIV prevalence, incidence and ART coverage (24–26). Kenya has a very wide range of HIV prevalence, from <0.1% in eastern regions to >25% in its western regions (27). Accordingly, this analysis focused only on the high-prevalence Nyanza region in western Kenya, composed of the six counties of Homa Bay, Kisii, Kisumu, Nyamira, Migori, and Siaya. These three settings of western Kenya, South Africa, and Zimbabwe were selected based on high need, potential demand (28), enabling policies, regulatory environments and high HIV prevalence (Supplementary Material Table S1 in Supporting Information).

Model calibration to HIV epidemic trends in each setting was performed by varying sexual behavior parameters using parallel simultaneous perturbation optimization, a form of stochastic gradient descent designed for parallel computing (29, 30). Among all model parameter combinations tested, we selected 250 parameter sets that best fit epidemic trends using a roulette sampling technique (31).

### DPP intervention assumptions

DPP scale-up scenarios (Table 1) were designed with input from the DPP Consortium (32), a collaboration of researchers, funders, advocates, and prospective implementers, including experts from both HIV prevention and family planning (33). In our main analysis, we simulated DPP provision to current OCP users ages 25 to 49, in whom uptake and adherence rates to OCPs and oral PrEP are generally higher than in adolescent girls and young women (AGYW) (34). We assumed DPP adherence would be equivalent to OCP adherence, leading to no change in pregnancy risk and a 90% reduction in HIV risk (Scenario 1). We additionally simulated DPP provision to alternative populations: AGYW ages 15 to 24 years (Scenario 2), female sex workers (FSW, Scenario 3), and HIV-negative women in stable serodiscordant couples (Scenario 4). Because there is no available data on real-life DPP use and it is not yet known how the DPP will impact adherence, we also analyzed a range of alternative DPP adherence pattern leading to HIV and pregnancy prevention effectiveness between 30% and 95% (Scenarios 5 through 8). We refer to these risk reduction rates as “effective protection” because they are intended to reflect the variable effectiveness rates that would result from different use patterns and adherence rates. While it is hypothesized that the DPP may increase adherence, assessing outcomes with more pessimistic assumptions around effective protection is important to understanding the potential impact across a wide range of use scenarios.

**Table 1:** Scenarios in which DPP cost-effectiveness was analyzed (scenario 1 serves as a primary analysis). Each scenario was run for Kenya, Zimbabwe, and South Africa.

Scenario	Population	DPP Effective Protection for both HIV Pregnancy*	Comparison scenario
1	Ages 25–49	90%	OCP users (assumed receive 90% effective protection against pregnancy with no effect on HIV acquisition)
2	Ages 15–24		
3	Sex workers		
4	Serodiscordant		
5	Ages 25–49	30%	
6		61%	
7		73%	
8		95%	
9	Ages 25–49	90%	Unmet need to contraception (no effect on HIV or pregnancy)
10	Ages 25–49	90%	Condom users (90% effective protection against pregnancy, 80% effective protection against HIV)
11	Ages 25–49	90%	PrEP with 73% effective protection against HIV (no effect on pregnancy)
12	Ages 25–49	90%	PrEP with 73% effective protection against HIV, plus OCP with 90% effective against pregnancy
13	Ages 25–49	90%	Same as Scenario 1 modeled over a 20-year time horizon
14	Ages 25–49	90%	Same as Scenario 1 modeled over a 40-year time horizon
15	Ages 25–49	90%	Same as Scenario 1 analyzed with a 0% annual discount rate
16	Ages 25–49	90%	Same as Scenario 1 analyzed with a 6% annual discount rate

* Reduction in HIV acquisition risk as a result of different patterns of DPP adherence. The difference between this number and 90% is additional used to model the differential risk of unintended pregnancy as described in Methods.

### Counterfactual assumptions

Counterfactual assumptions were used to determine the scenario against which each DPP scenario was compared in order to assess incremental health impacts and costs. In most of our analyses (Scenarios 1 through 8 and 11 through 16) we assumed that, in the absence of DPP, users would instead use OCP with typical use, with a 90% lower annual risk of pregnancy compared to having unmet need for contraception (35, 36). Other counterfactual assumptions included having unmet need for contraception (Scenario 9), using male condoms (assuming 75.5% effectiveness against pregnancy and 80% effectiveness against HIV, Scenario 10) (35, 36), using PrEP (assuming 73% reduction in HIV risk, Scenario 11), and delivering both PrEP and OCP simultaneously (with 73% HIV risk reduction and 90% pregnancy risk reduction, Scenario 12).

### Reproductive health assumptions

For analyses in which the counterfactual included unmet need for contraception (Scenarios 9 and 11), a less effective form of contraception (Scenario 10), or differential adherence to contraception with the DPP vs. OCP (Scenarios 5 through 8), we incorporated health effects of increased or decreased rates of unintended pregnancy. For women with unmet need for contraception, unintended pregnancy was assumed to occur at an annual rate of 34%, accounting for lower observed fertility among sexually active women who do not desire pregnancy compared to women who desire pregnancy, even when no method is used (37). Pregnancy risk reduction was applied to this baseline rate, e.g., OCP with typical use resulted in a 3.4% annual risk of pregnancy (35, 36).

We calculated disability-adjusted life-years (DALYs) caused by HIV or unintended pregnancy by factoring in years of life lost (YLLs) and years lived with disability (YLDs). Disability weights and life expectancies used can be found in Supplementary Material Tables S2 and S3 in the Supporting Information. DALYs averted were calculated as the difference between the DALYs with DPP rollout and the counterfactual. Pregnancy-related mortality rate associated to unintended pregnancy were calculated using the values in Table 3 with the equation:

$$\begin{array}{rl} & P_{\;preg}(F_{livebirth}M_{livebirth}+F_{abortion}M_{abortion}+F_{miscarriage}M_{miscarriage}\\ & +F_{stillbirth}M_{stillbirth}) \end{array}$$

where $P_{\;preg}$ is the annual probability of becoming pregnant, $F_{livebirth}$ is the proportion of unintended pregnancies ending in live birth, $M_{livebirth}$ is the maternal mortality rate associated with live birth with an unintended pregnancy, $F_{abortion}$ is the proportion of unintended pregnancies ending in abortion, $M_{abortion}$ is the abortion mortality rate, $F_{miscarriage}$ is the proportion of unintended pregnancies ending in miscarriage, $M_{miscarriage}$ is the maternal mortality rate from miscarriage, $F_{stillbirth}$ is the proportion of pregnancies ending in stillbirth, and $M_{stilbirth}$ is the maternal mortality rate from stillbirth.

### Cost assumptions

We estimated the net cost to the healthcare system of each DPP implementation scenario relative to its corresponding counterfactual. Costs included the commodity and delivery costs of contraceptive and PrEP products (Table 2) as well as health care costs associated with HIV infection (Table 2) and unintended pregnancy (Table 3). All costs are reported in 2021 USD and accrued over a 30-year time horizon with 3% annual discounting. In sensitivity analysis, costs were accrued over a 20- or 40-year time horizon with a 0% or 6% annual discount rate (Scenarios 13–16).

**Table 2:** Assumptions for costs (2021 USD) of different HIV and contraceptive products based on contraceptive data from riley T et al. and Jamieson et al. (4, 38) and cost of goods sold (COGS) estimates from the Clinton Health Access Initiative. We assumed co-delivery of PrEP and OCP would reduce total delivery costs by 6% compared to separate delivery (39).

DPP provision (per person-year)	Cost
First year of use, 2025–2027	$166
Subsequent years of use, 2025–2027	$145
First year of use, 2028+	$146
Subsequent years of use, 2028+	$125
Oral PrEP provision (per person-year)	
First year of use, 2025–2027	$135
Subsequent years of use, 2025–2027	$114
First year of use, 2028+	$122
Subsequent years of use, 2028+	$101
OCP	
Per person-year	$12.5
Condoms	
Per person-year	$2.46
ART	
Per person-year	$257

**Table 3:** Assumptions for costs (2021 USD) and outcomes of unintended pregnancy based on pregnancy and maternal health outcome data from riley T et al (4) and delivery and abortion costs adapted from johns et al (40) and lince-deroche et al (41, 42) with adjustments based on country-specific personnel costs from riley T et al (4).

Health outcome	% of pregnancies	Associated costs	Maternal deaths/100,000 births or abortions
Live birth	49.6%	Kenya: $74 South Africa: $138 Zimbabwe: $86	Kenya: 391 South Africa: 140 Zimbabwe: 391
Miscarriage	11.9%		
Stillbirth	1.7%		
Induced abortion (safe)	9.2%	Kenya: $76 South Africa: $114 Zimbabwe: $89	Kenya: 152 South Africa: 26 Zimbabwe: 152
Induced abortion (less safe)	10.0%	Kenya: $89 South Africa: $125 Zimbabwe: $108	
Induced abortion (least safe)	17.6%	$0	

### Cost-effectiveness calculations

For each scenario, we generated model outputs of disability-adjusted life-years (DALYs) (43, 44), HIV infections averted, pregnancies averted, and costs. Net cost included DPP provision cost minus the cost of the alternative treatment (OCP or PrEP, if using), and minus maternal health and HIV treatment costs avoided through averted pregnancies and HIV infections. We generated incremental cost-effectiveness ratio (ICER) (45, 46) as follows:

$$\begin{array}{rl} & \mathrm{ICER}=\\ & \;\frac{\begin{array}{c} \mathrm{DPP}\;\mathrm{cost}-\mathrm{avoided}\;\mathrm{OCP}\;\mathrm{cost}-\mathrm{avoided}\;\mathrm{PrEP}\;\mathrm{cost}\\ -\;\mathrm{avoided}\;\mathrm{pregnancy}\;\mathrm{costs}-\mathrm{avoided}\;\mathrm{HIV}\;\mathrm{treatment}\;\mathrm{costs} \end{array}}{\begin{array}{c} \mathrm{DALYs}\;\mathrm{averted}\;\mathrm{due}\;\mathrm{to}\;\mathrm{HIV}\;\mathrm{prevention}\\ +\;\mathrm{DALYs}\;\mathrm{averted}\;\mathrm{due}\;\mathrm{to}\;\mathrm{pregnancy}\;\mathrm{prevention} \end{array}} \end{array}$$

In the main analysis, we analyzed the outcomes over a 30-year time horizon with 3% annual discount rate. In sensitivity analysis, we tested time horizons of 20 and 40 years, and discount rates of 0% or 6% annual discounting. To generate confidence intervals, we conducted bootstrap resampling from 250 repeated simulation runs for each scenario and its respective counterfactual.

## Results

### Impact of the DPP on HIV infections and pregnancies

**Figure 1:**
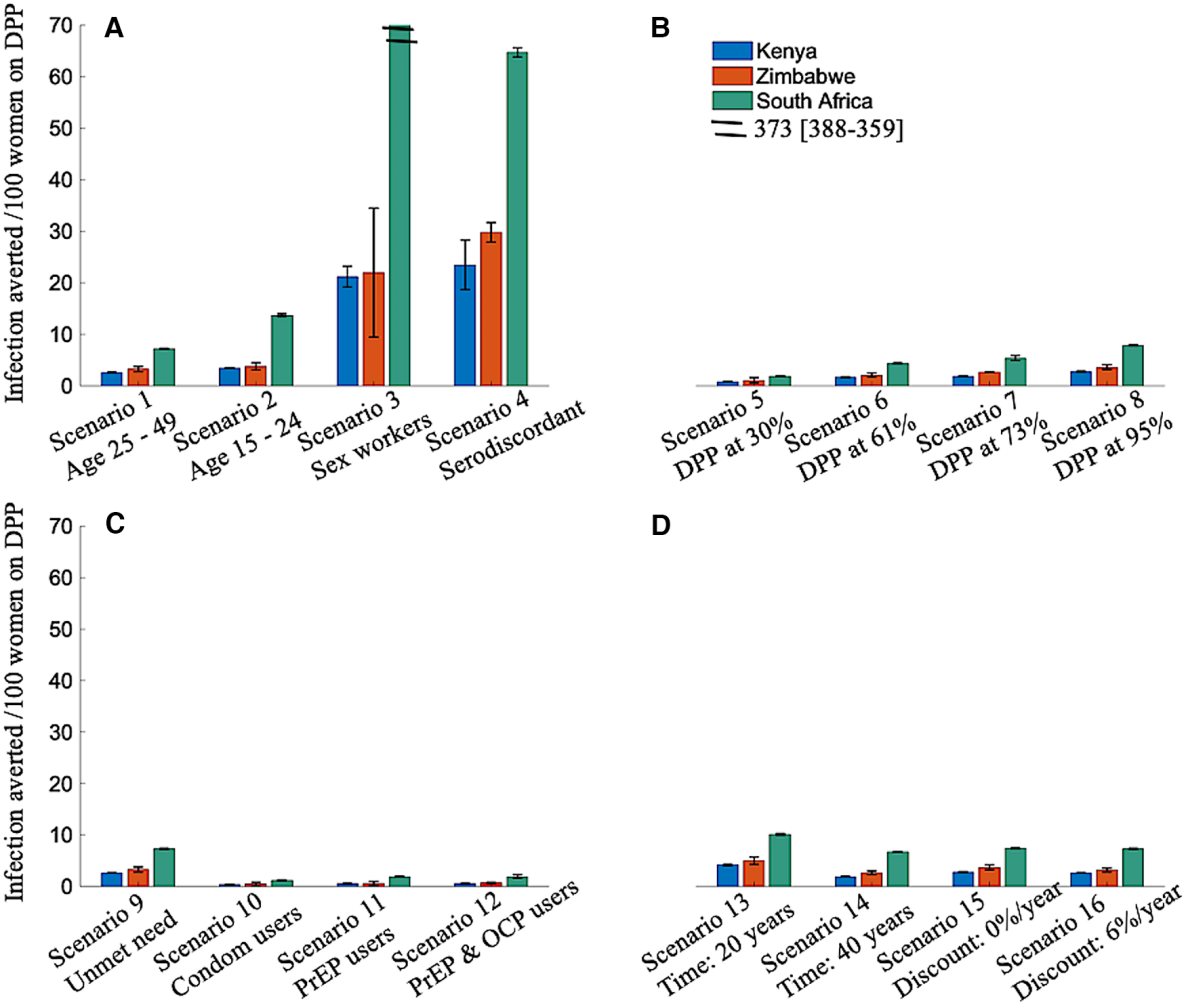
HIV infection averted per 1000 people on DPP across different populations (**A**), effective protection based on different adherence levels (**B**), alternative methods for HIV and pregnancy prevention (**C**), and time horizons and discount rates (**D**)

The number of HIV infections that could be averted per DPP user, in a scenario where this user would otherwise would not use PrEP, was lowest in Nyanza, Kenya and highest in South Africa (Figure 1), a reflection of the differences in HIV incidence across these settings (Supplementary Material Table S1). The number of infections averted was relatively modest among current OCP users with ages 25 to 49 (Figure 1) The highest number of infections averted, across all groups analyzed, was among female sex workers in South Africa, with an estimated 358.7–386.9 infections averted per 1,000 users per year. In Kenya and Zimbabwe, the largest number of infections averted was among women in stable serodiscordant couples, with 52.9–60.8 infections averted per 1,000 users per year in Kenya and 25.8–44.2 infections averted per 1,000 users per year in Zimbabwe.

In scenarios in which the DPP increased contraceptive use, unintended pregnancies averted were also substantial (Table 4). Among women with unmet need for contraception, the DPP could avert on average 225 pregnancies per 1,000 users per year in all settings. Among condom users, DPP could avert on average 36 pregnancies per 1,000 users per year. Among OCP users with ages 25 to 49, if the DPP were to increase contraceptive adherence resulting in an increase of effective protection from 90% to 95%, it could avert 13 pregnancies per 1,000 users per year. On the other hand, if the DPP were to decrease contraceptive adherence leading to a decrease in effective protection from 90% to 73%, 61%, or 30%, it could lead to 43, 75, or 150 additional unintended pregnancies per 1,000 users per year.

**Table 4:** Number of pregnancies averted per 1,000 users per year in all settings.

Scenario	Contraceptive assumptions	Pregnancies averted per 1,000 users per year
1–4, 12–16	DPP does not change contraceptive adherence	0
5	DPP reduces contraceptive effective protection from 90% to 30%	−150
6	DPP reduces contraceptive effective protection from 90% to 61%	−75
7	DPP reduces contraceptive effective protection from 90% to 73%	−43
8	DPP increases contraceptive effective protection from 90% to 95%	13
9, 11	Women with unmet need uptake DPP	225
10	Condom users will uptake DPP	36

### Net health impact of the DPP

**Figure 2:**
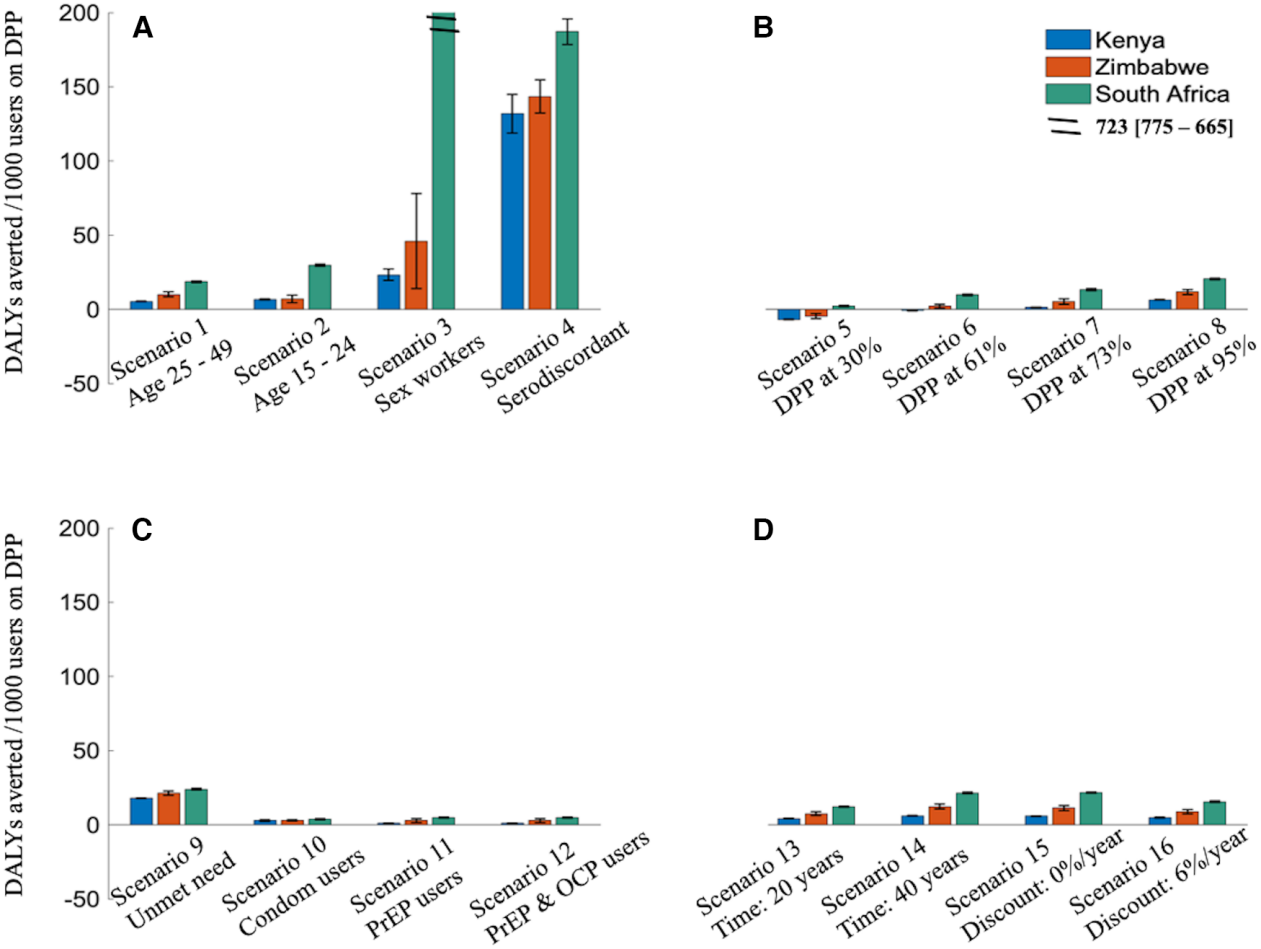
DALYs averted per 1,000 people on DPP across different populations (**A**), effective protection based on different adherence levels (**B**), alternative methods for HIV and pregnancy prevention (**C**), and time horizons and discount rates (**D**)

The net health impact, measured by DALYs averted, of the DPP's HIV and family planning effects was beneficial in most scenarios. The DPP was the most beneficial in sex workers and serodiscordant couples, whose risk of HIV was the highest, and among women with unmet need for contraception, for whom the DPP averted the most unintended pregnancies. In South Africa, the net benefit of the DPP was also high among adolescent girls and young women even if they would otherwise use OCP, on par with the benefit to older women with unmet need for contraception (Figure 2).

The DPP was estimated to be net harmful in a subset of settings and scenarios that explored potential reductions in contraceptive adherence and effective protection, relative to the use of OCP alone. Among OCP users ages 25–49 in Kenya, the DPP would be net harmful if efficacy effective protection against unintended pregnancy were to decline from 90% with OCP alone to 60% with DPP. In Zimbabwe, the DPP would still be net beneficial (but not cost-effective) with 60% effective protection, but would be net harmful with 30% effective protection. In South Africa, the DPP would be beneficial even with 30% effective protection because the health risks from HIV outweigh the risks of unintended pregnancy in this higher-incidence setting.

### Cost-effectiveness of the DPP

**Figure 3:**
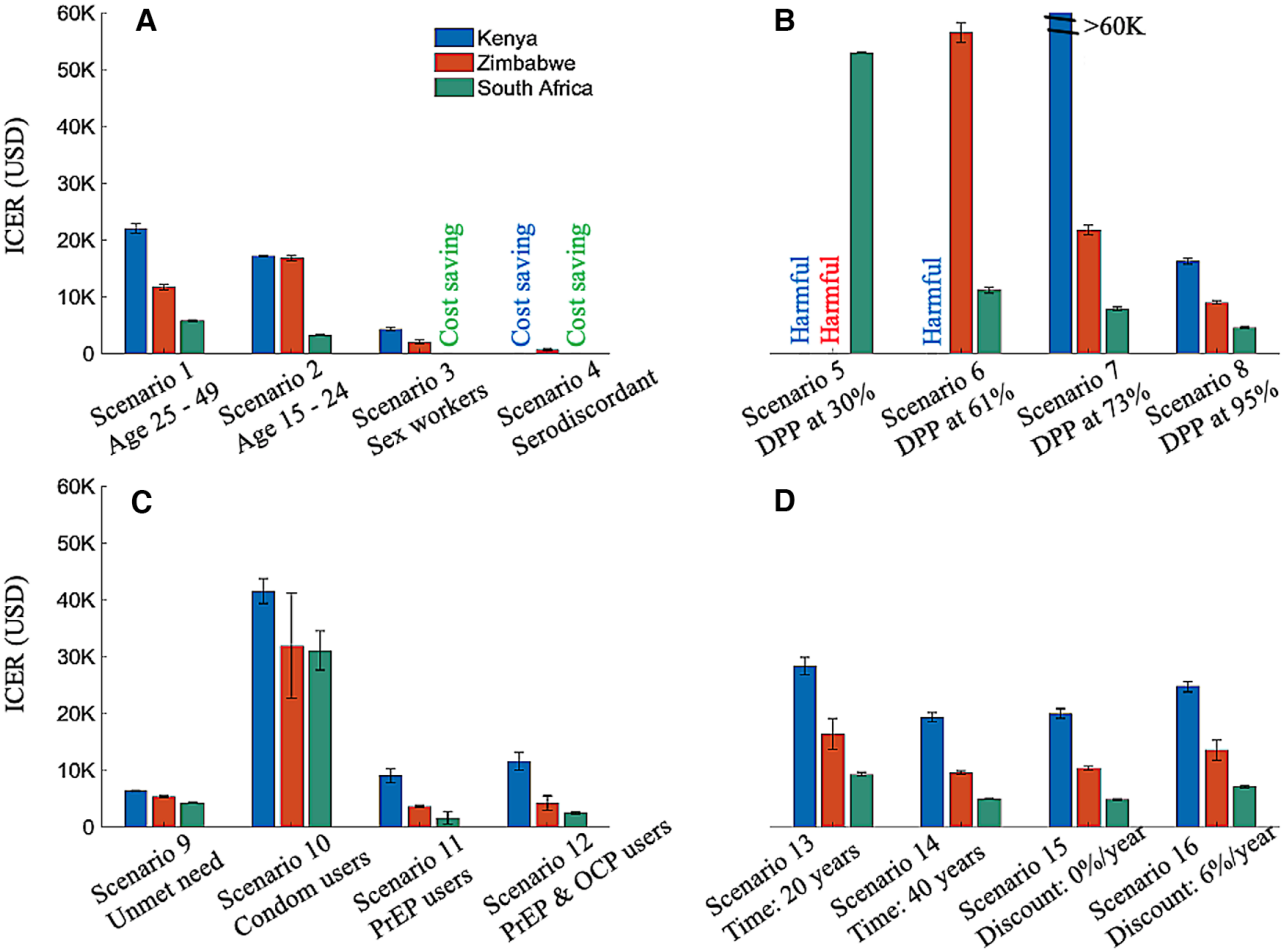
Cost-effectiveness of DPP across different populations (**A**), effective protection based on different adherence levels (**B**), alternative methods for HIV and pregnancy prevention (**C**), and time horizons and discount rates (**D**)

The cost-effectiveness of the DPP depended on HIV incidence in settings and populations where it would be implemented, with lower ICERs (greater cost-effectiveness) in the higher incidence setting of South Africa (green bars in Figure 3). Across all settings, the ICER of DPP was estimated to be in the thousands to tens of thousands of USD current OCP users ages 25–49 (Scenario 1) due to lower incidence compared to other population groups (Figure 3). Thresholds for cost-effectiveness are generally in the US$500–800 range for HIV services (47, 48), and lower for domestically-funded health services in low-income countries (49). Thus, it is not likely that the DPP will be cost-effective in older OCP users, even if adherence levels and effective protection are maintained when switching from the OCP to the DPP.

However, the DPP is more likely to be cost-effective for current in sex workers and women in stable serodiscordant couples, regardless of whether they currently use OCP, PrEP, both OCP or PrEP, or neither product. In these populations, the DPP averted substantial health systems costs by avoiding HIV treatment and obstetric costs. As a result, the DPP was not only beneficial and cost-effective, but was cost-saving among both sex workers and serodiscordant couples in South Africa, and among serodiscordant couples in Kenya. In Zimbabwe, the DPP was not cost-saving over a 30-year time horizon but was potentially cost-effective among serodiscordant couples (ICER = US$642, 95% CI: $432-$988).

The DPP is likely to a cost-effective alternative for PrEP users who are concurrently using OCP or have unmet need for contraception, especially if their adherence and, therefore, effective protection improves on the DPP relative to PrEP alone (Figure 3, Scenarios 11 and 12). This is due to the relatively small cost differential between PrEP and DPP (Table 2), making it possible to obtain the benefits of the DPP at relatively low incremental cost.

Among women with unmet need for contraception, the DPP was beneficial, but was unlikely to be cost-effective (ICER >$4,000 per DALY averted). The DPP among condom users was even less cost-effective, given the partial protection against HIV and pregnancy from condom use alone. This is due to the relatively high cost of the DPP, despite its substantial health benefits as a contraceptive for those who would not otherwise use a highly effective contraceptive method.

Our findings were robust to changes in the time horizon of analysis (20 to 40 years) and annual economic discount rate (0% to 3%) (Figures 1, 2, 3, Panel D).

## Discussion

This study used agent-based mathematical modeling to estimate the cost-effectiveness of the DPP across different populations and use cases in Nyanza, Kenya, Zimbabwe, and South Africa, taking into account the health impacts and costs from HIV and pregnancy prevention. We found that the DPP could have wide-ranging health economic implications, from health benefits with potential for cost-savings (in female sex workers and serodiscordant couples), to benefits that are unlikely to be cost-effective (in OCP users ages 25–49), to a potential for net harm (in OCP users who substantially reduce adherence after switching to the DPP). These results reflect similar trends to those seen in recent oral PrEP modeling, where cost-effectiveness varies widely by population and geography (18).

While cost-effectiveness provides a critical input to understanding future intervention costs and impact, it is only one of the many considerations in this decision-making process. Experience with oral PrEP has demonstrated that narrowly focusing on risk may have unintended negative consequences, including perpetuating stigma (50). These learnings underscore the need to ensure decision-making based on cost-effectiveness is balanced with broader programmatic and social considerations. However, understanding the groups and sub-populations among whom the DPP is most likely to be cost-effective will remain a crucial input to informing investment decision-making and ensuring budgets are effectively allocated to meet program goals.

Our analysis suggests that the DPP could be a cost-effective, and in some cases cost-saving, method of expanding PrEP use among women at high risk of HIV but is unlikely to be a cost-effective method to expand contraceptive use among women with lower HIV risk, even in the context of relatively high rates of unintended pregnancy. The lack of cost-effectiveness among women with unmet need for contraception is driven in part by declining HIV incidence in the general population, and in part by the high cost of the PrEP component of DPP. Because the cost of family planning alone is much lower than the projected cost of the DPP, addressing SSA's high unmet need for contraception will likely require redoubled efforts to improve family planning access, with more selective DPP use among women with greater HIV risk.

Cost-effectiveness was highly dependent on the setting in which DPP would be implemented, with higher HIV incidence leading to greater cost-effectiveness. Of the three settings modeled, cost-saving was more likely among high-incidence populations in South Africa. Given declining incidence and progress toward treatment targets in many parts of SSA, the DPP may not be a cost-effective alternative to existing options for many of SSA's women of reproductive age. Our results suggest that a “one size fits all” strategy is unlikely to lead to efficient and effective use of the DPP, and guidelines around its use are likely to require setting-specific health analyses and program planning.

Despite potential benefits offered by DPP, our analysis suggests that switching from OCP use to DPP use could be net harmful in some populations and settings if adherence decreases substantially. This is because, depending on the level of HIV risk in a given population segment, the health risks from unintended pregnancy can in some cases outweigh the health benefits from HIV prevention. On the other hand, the DPP may increase adherence among existing oral PrEP or OCP users due to the increased motivation to prevent both unintended pregnancy and HIV with a combined product. Our modeling demonstrates that this would lead to increased likelihood of cost-effectiveness. Careful monitoring, clear messaging, and effective counseling strategies will be critical to support informed choice among potential users. Future analyses could leverage forthcoming adherence data from clinical crossover studies to understand the implications of DPP adherence on risks and benefits for current OCP users.

### Limitations

Our analysis has several important limitations. First, we did not consider risk self-assessment (e.g., oral PrEP use concentrated into times of high-risk or multiple sexual partnerships). Evidence from oral PrEP suggests that users can time PrEP usage in risk-informed manners (38, 51–54). If this applies to DPP use, the DPP is likely more cost-effective than current analysis suggests. However, because the DPP is a dual indication product, it may not be suitable for users who would cycle on and off according to perceived risk from current partners, as usage patterns for contraception may not fully align with periods of risk for HIV acquisition. Ongoing risk-informed use could be explored in future research if determined to be relevant to DPP use patterns.

Second, we did not include incremental risks of neonatal mortality or child morbidity for children born as a result of unintended pregnancy. In our literature search, we found mixed results on the impact of unintended pregnancies on health outcomes of the child (55–57). In some cases, women were less likely to indicate a pregnancy was unwanted if it ended in neonatal death (57). Further study of health outcomes from unintended pregnancies are needed to quantify additional burdens due to putative increases in neonatal and child mortality and to socioeconomic burdens on individuals and society (58–61).

Third, we only considered DPP initiation among women using OCP, male condoms, or with unmet need for contraception. We did not consider alternative forms of contraception, ranging from less effective methods such as withdrawal, to more effective methods such as injections, implants, and intrauterine devices. Important questions remain about whether women currently using longer-acting forms of contraception would be recommended to use DPP, given than oral contraceptive methods tend to be less effective than longer-acting methods with typical use (62).

Fourth, our analysis only estimated DPP impact and cost-effectiveness in specific population segments and use cases, but did not estimate the total demand for DPP across the populations modeled, patterns of usage over the reproductive lifecourse, or the aggregate effect of DPP introduction on HIV and unintended pregnancy rates. Introduction of new contraceptive methods has generally tended to increase overall contraceptive use by meeting the needs and preferences of more users (14). Preliminary evidence from PrEP research suggests that expanded PrEP method mix may also increase overall use (63). However, the ability to receive and adherence to the DPP is likely to vary over the reproductive lifecourse due to factors including reproductive health knowledge, marital status, and pregnancy intentions. The effect of a dual-indication product such as DPP on overall coverage for each use case, and in aggregate over the lifecourse, is not currently known and warrants further research.

Fifth, we focused exclusively on the DPP and not other MPT products. At the time of writing, the DPP is the only MPT in late-stage development and appears likely to be the first MPT to reach markets since the male condom. However, it is worth noting that additional MPTs are in earlier stages of the discovery and development process, including injections, implants, and vaginal rings, films, and gels. As the landscape of viable MPT products becomes clearer, our analysis will require revision to account for potential product alternatives, an indeed a possible array of MPT method options offering women more choices than the DPP alone.

Finally, like all models, our model is a simplification of a complex process. We attempted to capture important aspects of HIV and unintended pregnancy, but our results are only an approximation of heterogeneous populations and health risks. Results should be used with caution and in context, and updated as new evidence accrues.

## Conclusion

With the potential to be the first MPT for HIV and pregnancy to be introduced since male and female condoms, the DPP has the potential to provide significant health benefits for some groups of women. The DPP is most likely to be cost-effective among populations at high HIV risk or as an alternative to oral PrEP use with or without concurrent OCP use, and it may be cost-saving in some populations and settings with particularly high HIV incidence. Effective counseling and decision-making tools for prospective users will be important, as outcomes are sensitive to adherence.

## Data Availability

The original contributions presented in the study are included in the article/**Supplementary Material**, further inquiries can be directed to the corresponding author.
