# Supplementary material for: Cost-effectiveness of the dual prevention pill for contraception and HIV pre-exposure prophylaxis
Source: Front Reprod Health. 2023 May 17;5:1144217. doi: 10.3389/frph.2023.1144217 (PMC10230827; doi:10.3389/frph.2023.1144217)
Supplement: Supplementary file 1 [file Datasheet1.docx]

**Supporting information**

**Cost-effectiveness of the Dual Prevention Pill for Contraception and HIV Pre-Exposure Prophylaxis**

**Masabho P. Milali^1^, Danielle Resar^2^, David Kaftan^1^, Jennifer Campbell^2^, Adebanjo Olowu^2^, Danny Edwards^2^, Ingrida Platais^1^, Hae-Young Kim^1^, Sarah Jenkins^2^, Anna Bershteyn^1^**

^1^Department of Population Health, NYU Grossman School of Medicine, New York, NY, USA

^2^Clinton Health Access Initiative, Boston, MA, USA

***Correspondence:**

Masabho Peter Milali

Masabho.milali@nyulangone.org

**Supplementary Tables**

**S1 Table:** HIV Prevalence (%) in study countries as of 2021(S1)

| **Population** | **Nyanza, Kenya** | **Zimbabwe** | **South Africa** |
| --- | --- | --- | --- |
| Adult aged 15-49 | 11.3 [9.7 – 12.9] | 11.6 [10.2 – 12.7] | 18.3 [15.6 - 20.5] |
| Women aged 15-49 | 14.8 [12.7 – 16.9] | 14.4 [12.6 – 15.9] | 24.5 [20.8 - 27.7] |
| Men aged 15-49 | 7.8 [6.7– 8.9] | 8.7 [7.0 – 9.8] | 12.1 [9.5 - 13.6] |

**S2 Table**: Disability Weights according to GBD Survey (S2)

| **Health State** | **DALY Weight** |
| --- | --- |
| HIV Negative | 0 |
| HIV Positive, not on ART | 0.274 |
| HIV Positive, on ART | 0.078 |
| Dead | 1.0 |

**S3 Table:** Life expectancies from age 15 for each setting (S3)

| Country | Life Expectancy |
| --- | --- |
| South Africa | 51.4 |
| Zimbabwe | 49.6 |
| Kenya | 54.9 |

**S4 Table:** DPP and oral PrEP annual provision cost components, 2021 USD inflation adjusted.

| Row | Component | Nyanza, Kenya | Zimbabwe | South Africa | Source |
| --- | --- | --- | --- | --- | --- |
| A | OCP commodity costs | $4.89 | $4.89 | $4.29 | Guttmacher (2019) (S4) |
| B | PrEP commodity costs | $47.78 | $47.78 | $45.43 | Jamieson (2020)  (S5) |
| C | DPP commodity costs before reaching scale (2025-2027) | $75.00 | $75.00 | $75.00 | CHAI Study (proprietary) |
| D | DPP commodity costs at scale (2028+) | $55.00 | $55.00 | $55.00 | CHAI Study (proprietary) |
| E | OCP non-commodity costs | $8.03 | $8.03 | $8.30 | Guttmacher (2019) (S4) |
| F | PrEP non-commodity costs, first year | $88.73 | $88.73 | $84.36 | Jamieson (2020)  (S5) |
| G | PrEP non-commodity costs, subsequent years | $65.98 | $65.98 | $62.73 | Jamieson (2020)  (S5) |
| H | Male condom provision | $2.46 | $2.46 | $2.46 | Guttmacher (2019)  (S4) |
| I | Non-commodity cost savings for integrated PrEP/OCP | 6% | 6% | 6% | Hewett (2016)(S6) |
| J | DPP first year of use, 2025-2027 | $165.95 | $165.95 | $162.10 | (1-J)*(E+F) + C |
| K | DPP subsequent years of use, 2025-2027 | $144.57 | $144.57 | $141.77 | (1-J)*(E+G) + C |
| L | DPP first year of use, 2028+ | $145.95 | $145.95 | $142.10 | (1-J)*(E+F) + D |
| M | DPP subsequent years of use, 2028+ | $124.57 | $124.57 | $121.77 | (1-J)*(E+G) + D |
| N | Oral PrEP first year of use, 2025-2027 | $135.15 | $135.15 | $128.49 | F + B |
| O | Oral PrEP subsequent years of use, 2025-2027 | $113.76 | $113.76 | $108.16 | G + B |
| P | Oral PrEP first year of use, 2028+ | $122.41 | $122.41 | $116.38 | F + B*(D/C) |
| Q | Oral PrEP subsequent years of use, 2028+ | $101.02 | $101.02 | $96.04 | G + B*(D/C) |

**S5 Table:** Abortion Method Frequency from Guttmacher (2019) (S4)

| Category | Method | Nyanza, Kenya | Zimbabwe | South Africa |
| --- | --- | --- | --- | --- |
| Safe | Manual or electric vacuum aspiration | 60% | 60% | 45% |
|  | Dilation and evacuation | 10% | 10% | 10% |
|  | Mifepristone and misoprostol (<12 weeks/84 days) | 0% | 0% | 40.5% |
|  | Mifepristone and misoprostol (≥12 weeks) | 0% | 0% | 4.5% |
|  | Misoprostol (<12 weeks) | 27% | 27% | 0% |
|  | Misoprostol (≥12 weeks) | 3% | 3% | 0% |
| Less safe | Manual or electric vacuum aspiration | 15% | | |
|  | Dilation and evacuation | 25% | | |
|  | Misoprostol (<12 weeks) | 54% | | |
|  | Misoprostol (≥12 weeks) | 6% | | |

**S6 Table:** Health Outcomes of Unintended Pregnancy from Guttmacher (2019) (S4)

| outcome | Sub-Saharan Africa (SDG Region) |
| --- | --- |
| Live Birth | 49.61% |
| Safe abortion | 9.15% |
| Less safe abortion | 10.03% |
| Least safe abortion | 17.60% |
| miscarriage | 11.92% |
| stillbirth | 1.68% |

**S7 Table:** Unintended pregnancy cost components, 2021 USD inflation adjusted.

| component | Kenya | Zimbabwe | south africa | Source |
| --- | --- | --- | --- | --- |
| Nurse/midwife salary per minute | $0.05 | $0.08 | $0.26 | Guttmacher (2019)  (S4) |
| Delivery | $74 | $86 | $138 | Assumption based on Johns (2019), Guttmacher (2019)  (S4) |
| Induced Abortion (Safe) | $76 | $89 | $114 | Assumption based on Lince-Deroche (2017), Lince-Deroche et al., 2018 (S7), Guttmacher (2019)  (S4) |
| Induced Abortion (Less Safe) | $89 | $108 | $125 | Assumption based on Lince-Deroche (2017), Lince-Deroche (2018)(S7), Guttmacher (2019)  (S4) |
| Induced aboRtion (least Safe) | $0 | $0 | $0 | Guttmacher (2019)  (S4) |

**References**

S1. UNAIDS data 2021 [Internet]. [cited 2022 Nov 26]. Available from: https://www.unaids.org/en/resources/documents/2021/2021_unaids_data

S2. Global Burden of Disease Collaborative Network. Global Burden of Disease Collaborative Network. Global Burden of Disease Study 2019 (GBD 2019) Disability Weights [Internet]. Institute for Health Metrics and Evaluation (IHME); 2020 [cited 2022 Nov 26]. Available from: http://ghdx.healthdata.org/record/ihme-data/gbd-2019-disability-weights

S3. Knoema. World and regional statistics, national data, maps, rankings. [Internet]. [cited 2022 Nov 26]. Available from: https://knoema.com//atlas

S4. Guttmacher Institute, Riley T, Sully EA, Lince-Deroche N, Firestein L, Murro R, et al. Adding It Up: Investing in Sexual and Reproductive Health 2019—Methodology Report [Internet]. Guttmacher Institute; 2020 Jul [cited 2022 Nov 4]. Available from: https://www.guttmacher.org/report/adding-it-up-investing-in-sexual-reproductive-health-2019-methodology

S5. Jamieson L, Gomez GB, Rebe K, Brown B, Subedar H, Jenkins S, et al. The impact of self-selection based on HIV risk on the cost-effectiveness of preexposure prophylaxis in South Africa. AIDS [Internet]. 2020;34(6). Available from: https://journals.lww.com/aidsonline/Fulltext/2020/05010/The_impact_of_self_selection_based_on_HIV_risk_on.9.aspx

S6. Hewett PC, Nalubamba M, Bozzani F, Digitale J, Vu L, Yam E, et al. Randomized evaluation and cost-effectiveness of HIV and sexual and reproductive health service referral and linkage models in Zambia. BMC Public Health. 2016 Dec;16(1):1–19.

S7. Lince-Deroche N, Constant D, Harries J, Kluge J, Blanchard K, Sinanovic E, et al. The costs and cost effectiveness of providing second-trimester medical and surgical safe abortion services in Western Cape Province, South Africa. PLOS ONE. 2018 Jun 28;13(6):e0197485.
